# Complex Circulation of Foot-and-Mouth Disease Virus in Cattle in Nigeria

**DOI:** 10.3389/fvets.2020.00466

**Published:** 2020-08-20

**Authors:** Hussaini G. Ularamu, David J. Lefebvre, Andy Haegeman, Yiltawe S. Wungak, David O. Ehizibolo, David D. Lazarus, Annebel R. De Vleeschauwer, Kris De Clercq

**Affiliations:** ^1^FMD Laboratory, Viral Research Division, National Veterinary Research Institute (NVRI), Vom, Nigeria; ^2^Sciensano, Scientific Direction of Infectious Diseases in Animals, Service for Exotic Viruses and Particular Diseases, Brussels, Belgium

**Keywords:** foot-and-mouth disease virus, foot-and-mouth disease (FMD), Nigeria, VP1, phylogeny, topotypes

## Abstract

Nigeria is a large densely populated country in West Africa. Most of its livestock is raised in a pastoralist production system with typical long distance migration in search of water and feed. As the demand for animal products largely exceeds the domestic production, large numbers of livestock are imported from neighboring countries without sanitary restrictions. In Nigeria, foot-and-mouth disease virus (FMDV) serotypes O, A, and Southern African Territories (SAT)2 are endemic for a long time. Clinical outbreaks of FMD due to serotype SAT1 are described again since 2015, after an absence of more than 30 years. Historically, outbreaks of FMD due to serotypes O, A, SAT1, and SAT2 were each time associated with trade of cattle entering Nigeria from neighboring countries. In the present study, tissue samples from 27 outbreaks of FMD were collected in Nigerian cattle from 2012 until 2017 in six different States and in the Federal Capital Territory. FMDV was isolated and serotyped and further characterized by VP1 sequencing and phylogenetic analysis to gain more knowledge on FMDV circulation in Nigeria. Half of the outbreaks were characterized as FMDV topotype O/EA-3, while outbreaks with other serotypes and topotypes were—in descending order—less prevalent: A/Africa/G-IV, SAT1/X, SAT2/VII, and O/WA. The high dynamics and omnipresence of FMD in Nigeria were illustrated in Plateau State where FMDV serotypes O, SAT1, and SAT2 were isolated during the course of the study, while at some point in the study, outbreaks due to FMDV serotype A were observed in three remote States. The genetic and phylogenetic analysis suggests a mixed origin of FMD outbreaks. Some outbreaks seem to be caused by sustained local transmission of FMDV strains present in Nigeria since a number of years, while other outbreaks seem to be related to recent incursions with new FMDV strains. The role of African buffaloes in the etiology of FMD in Nigeria is unclear, and sampling of wildlife is needed. The results of the present study suggest that systematic sample collection is essential to understand the complex concomitance of FMDV strains in Nigeria and essential to support the implementation of a vaccination-based control plan.

## Introduction

Foot-and-mouth disease (FMD) is an acute viral infection in domestic and wild cloven-hooved animals. Viral replication causes fever and painful lesions in the mouth and on the feet resulting in lethargy, reduced feed intake, and lameness. Direct losses for farmers are due to reduced milk and meat production and to reduced draft power and transportation. Mortality may occur, usually in very young animals. Due to international trade restrictions, countries affected by FMD cannot export susceptible animals or products of these animals to countries free from FMD. As a result of direct losses and trade restrictions, FMD significantly contributes to food insecurity and poverty in endemic regions in Africa and Asia (1–3).

The foot-and-mouth disease virus (FMDV), an *Aphthovirus* in the family of the *Picornaviridae*, exists in seven different serotypes [A, O, C, Asia 1, Southern African Territories (SAT)1, SAT2, and SAT3]. It is a non-enveloped icosahedral virus consisting of four different structural proteins (VP1, VP2, VP3, and VP4) and a single-stranded positive sense RNA genome of ~8.5 kb, which also encodes a number of nonstructural proteins that are expressed in the host cell during viral replication. The VP1 protein contains the most important neutralizing epitope, and the VP1 gene is mostly used for phylogenetic characterization because of its biological relevance and its heterogeneity. The VP1 gene is very well suited for routine monitoring of transboundary movements of FMDV (1, 4, 5).

Nigeria is a large, densely populated country in West Africa (surface 924,000 km^2^, >200 million inhabitants) (6). Most of its agriculture is subsistence-oriented, and although there is an estimated population of 21 million cattle, 43 million sheep, 81 million goats, and 7 million pigs, the demand for meat products of these animal species largely exceeds the domestic production (7). Due to its low mortality compared to some other livestock diseases, FMD is not considered to be of the highest priority by Nigeria's competent authorities. Nevertheless, a recent study shows the high impact of FMD on the pastoral local dairy production system and on the food security and livelihood of the affected communities (8). There is no systematic surveillance for FMD in Nigeria and no control program, and only sporadic FMD notifications are made. Vaccination is not practiced except in a few established farms that have exotic cattle breeds which are more prone to severe clinical signs of FMD than domestic breeds (9). The Federal Republic of Nigeria is divided into 36 States plus the Federal Capital Territory (FCT) and, geopolitically, Nigeria is divided into six zones: North Central, North East, North West, South East, South South, and South West. About 90% of the cattle population and 70% of the sheep and goat populations are concentrated in the northern region of Nigeria (10).

FMD is considered endemic in domesticated livestock in Nigeria, and four different serotypes of FMDV are circulating at present: A, O, SAT1, and SAT2 (11–15). Remarkably, FMDV serotype SAT1 was never isolated from clinical cases of FMD in Nigeria in the period between 1981 and 2015 (12, 16). As in many other countries in sub-Saharan Africa, livestock movement is not controlled and FMD spreads in Nigeria due to unrestricted local and transboundary trade and due to the pastoral farming system which is characterized by long distance migration of livestock in search for greener pasture and watering points. Apart from trade practices and transhumance activities, other factors that contribute to the spread of FMD in Nigeria may be political conflict, which increases movements of nomadic people and their herds, conflict between arable farmers and pastoralists resulting from competition for resources, and the presence of game reserves as a possible source of infection for susceptible livestock (9, 13).

In the present study, samples from clinical cases of FMD in Nigerian cattle were collected from November 2012 until September 2017. The obtained FMDVs were characterized by virological and molecular techniques and compared to previously obtained and characterized viruses. As such, the aim of the study is to gain more in-depth knowledge on the circulation and distribution of FMDVs in Nigeria.

## Materials and Methods

### Clinical Specimens

**Figure 1:**
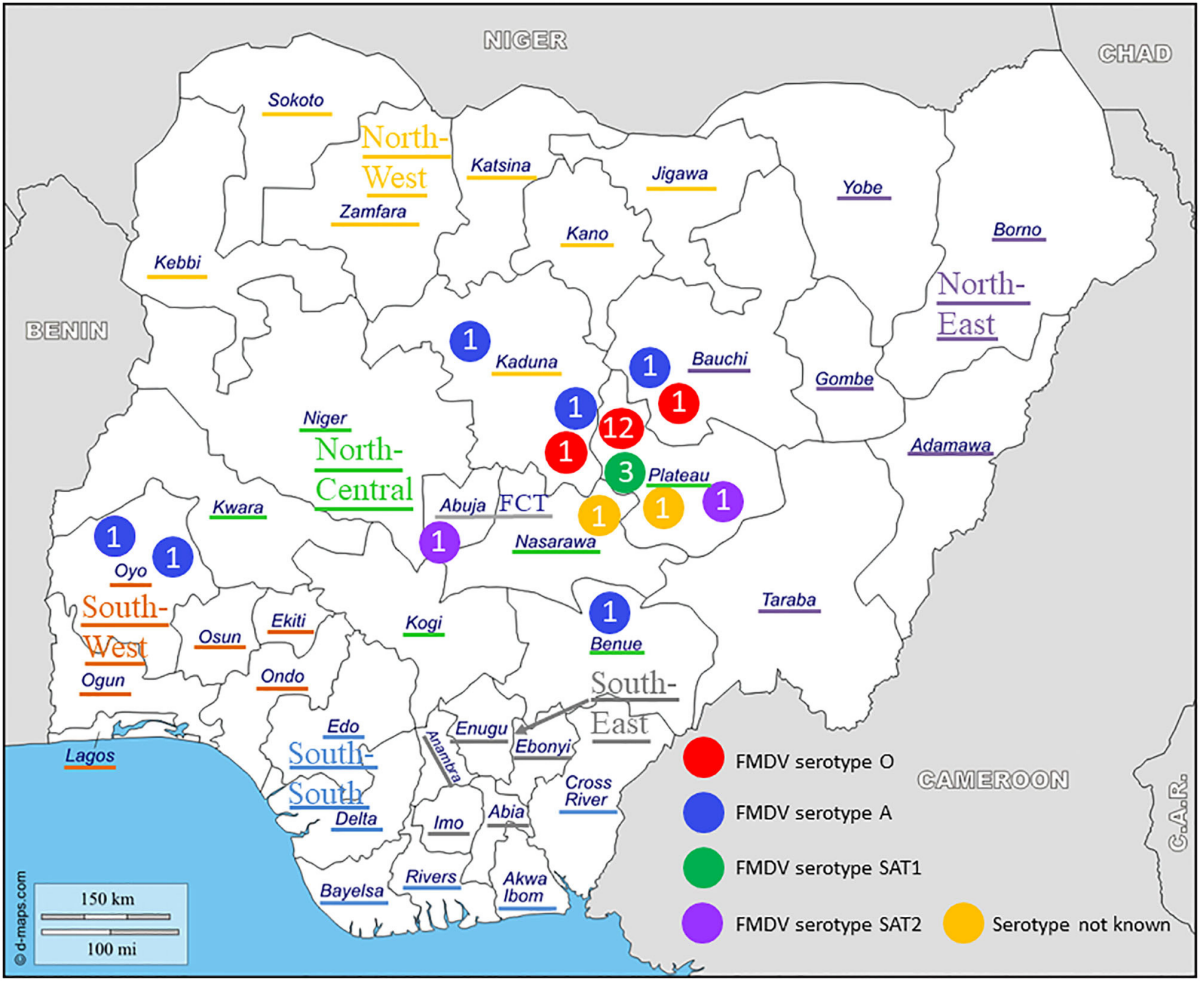
Geographical distribution and serotypes of foot-and-mouth disease virus (FMDV) isolates collected in Nigeria between 2012 and 2017. The geopolitical zones and the Federal Capital Territory (FCT) are indicated.

Epithelial tissue samples were collected from clinical cases of FMD in cattle in five States in Northern Nigeria (Bauchi, Benue, Kaduna, Nasarawa, and Plateau) within three geopolitical zones (North Central, North East, and North West), one State in South West Nigeria (Oyo) and in Abuja FCT between November 2012 and September 2017, as shown in Figure 1. The epithelial tissue samples were collected from un-ruptured and freshly ruptured vesicles and stored in vials containing in-house 5X-PSGA (penicillin, streptomycin, gentamycin, and amphotericin-B) diluted 1:1 with glycerol. Samples were transported on ice to the National Veterinary Research Institute (NVRI) and stored at −80°C until processing or shipment on dry ice to Sciensano. In both institutes, the samples were processed and FMDV was characterized based on the procedures described in the FMD Chapter of the World Organization for Animal Health (OIE) Manual of Diagnostic Tests and Vaccines for Terrestrial Animals (17).

### Primary Characterization

At NVRI, FMDV present in the tissue samples was characterized by virus isolation on the fetal goat tongue cell line ZZ-R 127 and a commercial antigen ELISA (IZSLER Biotech Laboratory, Brescia, Italy), as detailed previously (15). Thereafter, 81 duplicated tissue samples, originating from 27 outbreaks (17 in Plateau, three in Kaduna, two in Bauchi, two in Oyo, one in Abuja FCT, one in Benue, and one in Nasarawa), were selected and send to Sciensano for confirmatory analysis, VP1 sequencing, and phylogenetic characterization.

### Confirmatory Analysis

The duplicated tissue samples were confirmed to be positive for FMDV by RNA extraction and real-time RT-PCR (rRT-PCR) using the “3D” and “5′-UTR” reference methods of Callahan et al. (18) and Reid et al. (19) as detailed previously (11), but with the addition of 5'-tails to the FMDV-specific primers to enhance the detection of FMDV as described by Vandenbussche et al. (20, 21).

Subsequently, virus isolation on porcine kidney cell line IB-RS-2 or ovine epithelial cell line OA3T was performed as detailed previously (11). In case of a positive result, as determined by cytopathic effect (CPE) formation, the FMDV present in the cell culture supernatant was serotyped by an in-house antigen ELISA detecting all seven serotypes of FMDV, as described in the OIE Manual (17) and as detailed previously (11).

From those outbreaks where the virus isolation yielded negative results despite the presence of high to moderate amounts of FMD viral RNA in the tissue samples (Ct-values <30 as determined by rRT-PCR), attempts were made to rescue infectious FMDV from viral RNA. Briefly, BHK-21 cells (100 μl at a concentration of 5 × 10^6^ cells/ml) in PBS were mixed in duplicate with 1 or 10 μl of RNA, respectively, in a cuvette (0.2 cm) and subjected to the exponential decay pulse protocol at a voltage of 150 V, a capacitance of 250 μF, and a resistance of 400 Ohm using a BioRad Gene Pulser Xcell electroporation system. Following electroporation, 1 ml of preheated (37°C) growth medium [minimum essential medium (MEM) with 20 μg/ml gentamicin and 1 μg/ml Fungizone] supplemented with 5% fetal bovine serum (FBS) was added to the cuvette. Subsequently, the resuspended electroporated cells were transferred into a 24-well cell culture plate containing 1 ml of growth medium and incubated in a CO_2_ incubator at 37°C for 48–72 h and monitored daily for the formation of CPE. Further passages on cell culture were performed as detailed previously (11). In case of CPE, the FMDV was serotyped as described above.

### VP1 Sequencing and Phylogenetic Analysis

From those outbreaks from where virus could be isolated or rescued by *in vitro* electroporation, at least one sample was sequenced and used for phylogenetic analysis. Briefly, the complete VP1 genomic region of FMDV present in the original sample was amplified by RT-PCR as described by Ayelet et al. (22) and further processed, sequenced, and analyzed as described previously (11). The % VP1 nucleotide identity between isolates was calculated using the multiple sequence alignment tool in BLAST® at https://blast.ncbi.nlm.nih.gov/Blast.cgi. The phylogenetic evaluation of the obtained FMDV VP1 regions were performed after a best fit model analysis. The resulting Bayesian information criterion (BIC) values were compared in combination with the obtained tree topologies of reference sequences in order to select the most optimal phylogenetic settings across the four FMDV serotypes included in this study. The evolutionary history was inferred by using the maximum likelihood (ML) method based on the Tamura-Nei model (23) and the neighbor-joining (NJ) method (24). Bootstrap analysis (1,000 replicates) was carried out for both methods (25) whereby branches corresponding to partitions reproduced in <50% bootstrap replicates were collapsed. For the initial ML tree(s), the heuristic searches were obtained by applying the NJ method to a matrix of pairwise distances estimated using the maximum composite likelihood (MCL) approach. A discrete Gamma distribution was used to model evolutionary rate differences among sites. All positions with <95% site coverage were eliminated. All calculations were performed in the MEGA6 software package (26). For the NJ analyses, the evolutionary distances were computed using the Tamura-Nei method (23) and the tree length was measured in substitutions per site. The rate variation among sites was modeled with a Gamma distribution. All ambiguous positions were removed for each sequence pair. Similar to the ML method, all calculations were done in MEGA6.

## Results

### Virus Identification and Confirmation

At Sciensano, all 81 samples received from NVRI were confirmed to be positive for FMDV by rRT-PCR. Virus could be isolated from 55 out of 81 samples originating from 24 out of 27 outbreaks and from one more outbreak by *in vitro* electroporation. The presence of four different serotypes of FMDV in the set of samples (O, A, SAT1, and SAT2) was confirmed by the in-house antigen ELISA. The results of the different obtained FMDV sequences are summarized in Table 1.

**Table 1:** Summary of foot-and-mouth disease virus sequences from Nigeria obtained in the present study.

**Sample ID**	**State**	**Local Government Area**	**Collection date (d/m/y)**	**FMDV serotype/topotype**	**GenBank accession #**
O/NIG/8/2013	Kaduna	Jema'a	27/06/2013	O/WA	MT239372
O/NIG/9/2013	Kaduna	Jema'a	27/06/2013	O/WA	MT239373
O/NIG/13/2014	Plateau	Jos South	23/03/2014	O/EA-3	MT239374
O/NIG/14/2014	Plateau	Jos South	09/06/2014	O/EA-3	MT239375
O/NIG/15/2014	Plateau	Jos South	18/07/2014	O/EA-3	MT239376
O/NIG/16/2014	Plateau	Barkin Ladi	20/07/2014	O/EA-3	MT239377
O/NIG/2/2015	Plateau	Jos South	14/09/2015	O/EA-3	MT239378
O/NIG/3/2015	Plateau	Jos South	14/09/2015	O/EA-3	MT239379
O/NIG/1/2017	Plateau	Barkin Ladi	03/08/2017	O/EA-3	MT185923
O/NIG/2/2017	Bauchi	Toro	05/08/2017	O/EA-3	MT185919
O/NIG/3/2017	Plateau	Jos South	25/08/2017	O/EA-3	MT185922
O/NIG/4/2017	Plateau	Jos South	28/08/2017	O/EA-3	MT185921
O/NIG/5/2017	Plateau	Mangu	31/08/2017	O/EA-3	MT185918
O/NIG/6/2017	Plateau	Jos-East	26/09/2017	O/EA-3	MT185920
A/NIG/08/2015	Bauchi	Toro	11/09/2015	A/Africa/G-IV	MG712579
A/NIG/09/2015	Bauchi	Toro	11/09/2015	A/Africa/G-IV	MT211640
A/NIG/01/2017	Oyo	Saki West	22/03/2017	A/Africa/G-IV	MT211641
A/NIG/02/2017	Benue	Makurdi	29/05/2017	A/Africa/G-IV	MT220002
A/NIG/04/2017	Kaduna	Kaura	04/07/2017	A/Africa/G-IV	MT228048
A/NIG/05/2017	Kaduna	Kaduna	21/09/2017	A/Africa/G-IV	MT228049
SAT1/NIG/5/2015	Plateau	Jos South	21/09/2015	SAT1/X	MT239384
SAT1/NIG/6/2015	Plateau	Jos South	27/11/2015	SAT1/X	MT239380
SAT1/NIG/7/2015	Plateau	Jos South	27/11/2015	SAT1/X	MT239381
SAT1/NIG/8/2015	Plateau	Jos South	02/12/2015	SAT1/X	MT239382
SAT2/NIG/7/2013	Abuja FCT	Abuja FCT	03/01/2013	SAT2/VII	MT239385
SAT2/NIG/1/2017	Plateau	Langtang North	14/09/2017	SAT2/VII	MT239383

### Serotype O

**Figure 2:**
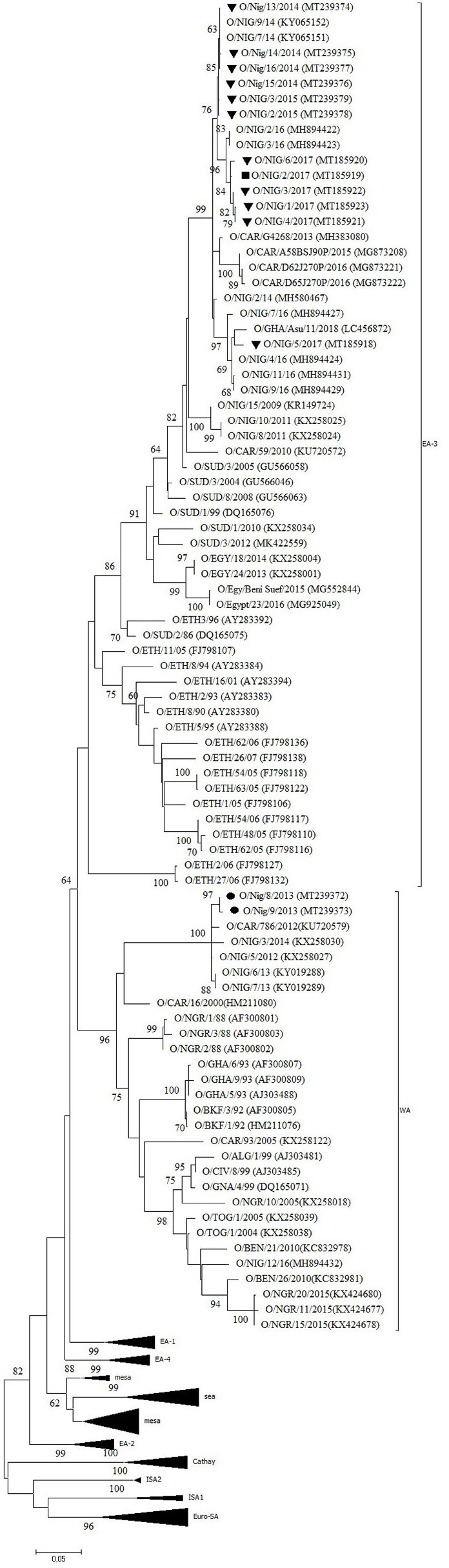
VP1 phylogenetic tree for foot-and-mouth disease virus (FMDV) serotype O inferred using the maximum likelihood method based on the Tamura-Nei model. Branch lengths indicate the number of substitutions per site. Bootstrap values ≥60% are indicated at the nodes. Novel Nigerian FMDV from this study from Plateau is indicated with ▼, from Kaduna with • and from Bauchi with ■.

Twelve outbreaks (four in Plateau State in 2014, two in Plateau in 2015, and five in Plateau and one in Bauchi in 2017) were characterized by antigen ELISA as FMDV serotype O and by VP1 sequencing and phylogenetic analysis as topotype O/EA-3. One more outbreak in Plateau (2014) was serotyped as O, but a valid nt sequence could not be obtained. In the NJ and ML phylogenetic trees (Figure 2), the isolates from 2014 and 2015 clustered with other Nigerian isolates from 2014 (11). Most of the isolates from 2017 clustered with Nigerian isolates from 2016 (14) and grouped together with the majority of the Nigerian isolates from 2014 and 2015. Within this group, we observed ~1% of difference in VP1 nt identity per year.

One isolate from Plateau (2017) clustered with other Nigerian isolates from 2016 (14) and with an isolate from Ghana (GHA, 2018), another country in West Africa, and with an isolate from 2014 from the Kachia Grazing Reserve in the neighboring Kaduna State, i.e., O/NIG/2/14 (13). Between this isolate from Plateau (2017) and the other isolates from 2017, we observed ~95% VP1 nt identity.

The two isolates from an outbreak in Kaduna State (2013) were characterized by antigen ELISA as FMDV serotype O and by VP1 sequencing and phylogenetic analysis as topotype O/WA and clustered with other contemporary O/WA isolates from Nigeria and neighboring country Cameroon (CAR) (11, 15, 27).

### Serotype A

One outbreak in Bauchi State in 2015 and four outbreaks in 2017 (two in Kaduna, one in Benue, and one in Oyo) were characterized by antigen ELISA as FMDV serotype A and by VP1 sequencing and phylogenetic analysis as topotype A/Africa lineage G-IV. One more outbreak in Oyo (2017) was serotyped as A, but a valid nt sequence could not be obtained. In the antigen ELISA, a frequent cross-reaction with serotype SAT1 was observed; on a single occasion, a cross-reaction with serotype SAT3. VP1 genomic sequences other than serotype A were however not found.

**Figure 3:**
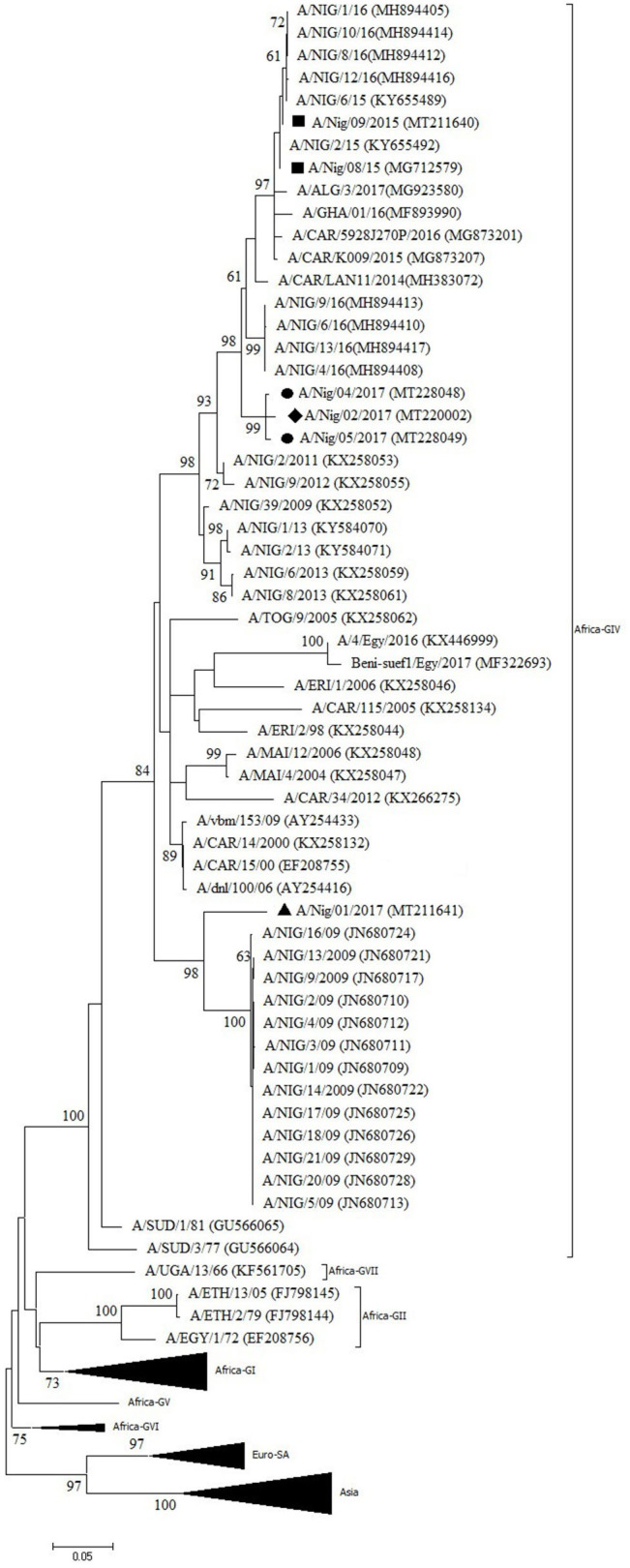
VP1 phylogenetic tree for foot-and-mouth disease virus (FMDV) serotype A inferred using the maximum likelihood method based on the Tamura-Nei model. Branch lengths indicate the number of substitutions per site. Bootstrap values ≥60% are indicated at the nodes. Novel Nigerian FMDV from this study from Kaduna is indicated with •, from Bauchi with ■, from Oyo with ▴ and from Benue with ♦.

In the NJ and ML phylogenetic trees (Figure 3), the serotype A isolates from Bauchi (2015) clustered with other Nigerian isolates from 2015 and 2016 (11, 14) with >99% VP1 nt identity. A highly similar VP1 nt identity was also observed with isolates from neighboring country CAR and GHA from 2015 and 2016 (28, 29). The isolates from Benue and Kaduna (2017) clustered separately and had 96% VP1 nt identity with Nigerian isolates from 2016 (14). The isolate from Oyo (2017) clustered with Nigerian isolates from 2009 (30) with 91% VP1 nt identity with these isolates. This isolate from Oyo (2017) had 87% VP1 nt identity with the isolates from Benue and Kaduna (2017).

### Serotype SAT1

**Figure 4:**
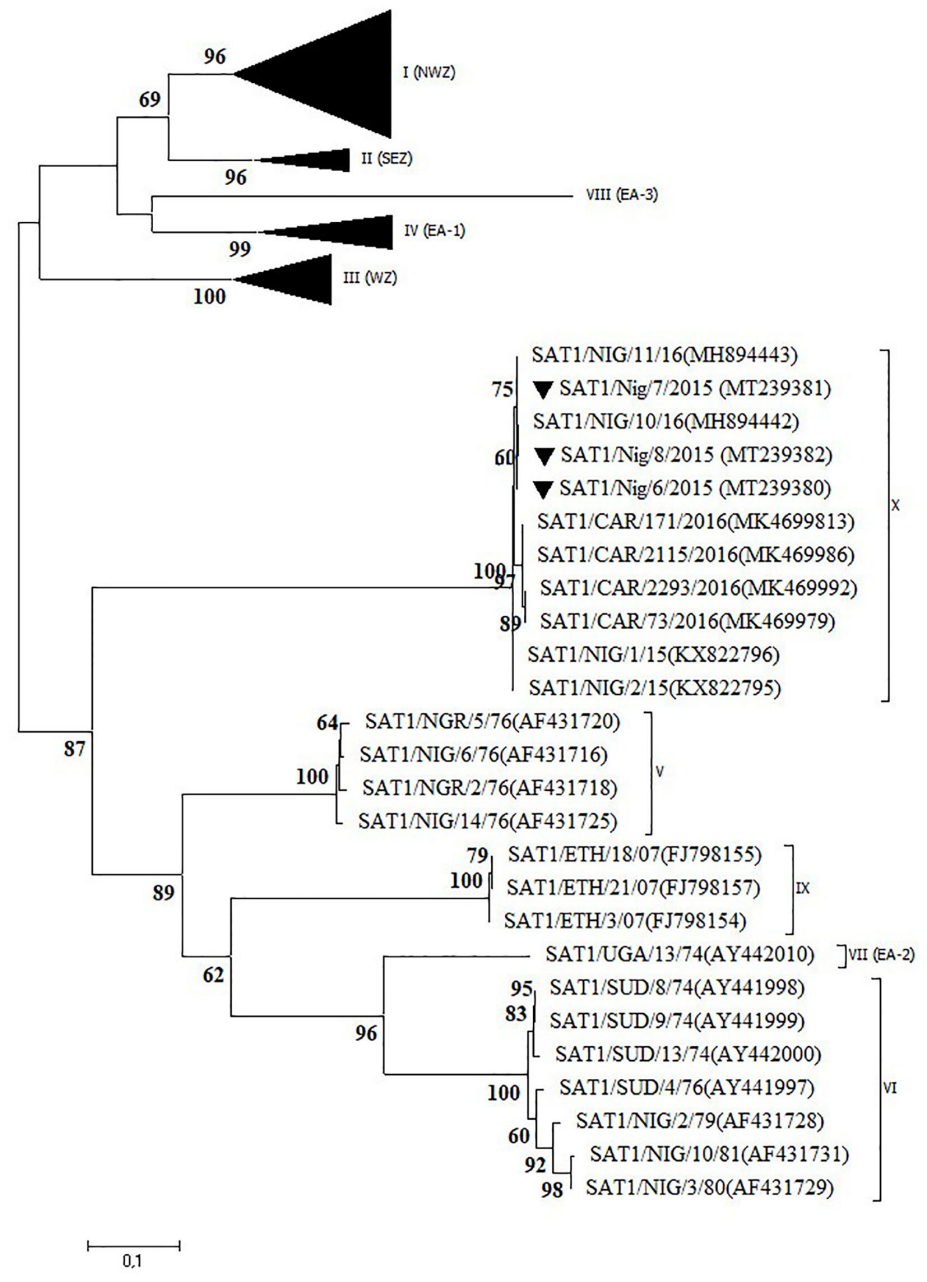
VP1 phylogenetic tree for foot-and-mouth disease virus (FMDV) serotype Southern African Territories (SAT)1 inferred using the maximum likelihood method based on the Tamura-Nei model. Branch lengths indicate the number of substitutions per site. Bootstrap values ≥60% are indicated at the nodes. Novel Nigerian FMDV from this study from Plateau is indicated with ▼.

Three outbreaks in Plateau State between September and December 2015 were serotyped as FMDV SAT1 and further characterized as topotype X. The isolates showed >99% VP1 nt identity with other Plateau isolates (2015–2016) and >98% VP1 nt identity with Cameroonian isolates from 2016 (12, 14). In the NJ and ML phylogenetic trees (Figure 4), the isolates from CAR clustered separately of those from Nigeria, as observed before (14).

### Serotype SAT2

**Figure 5:**
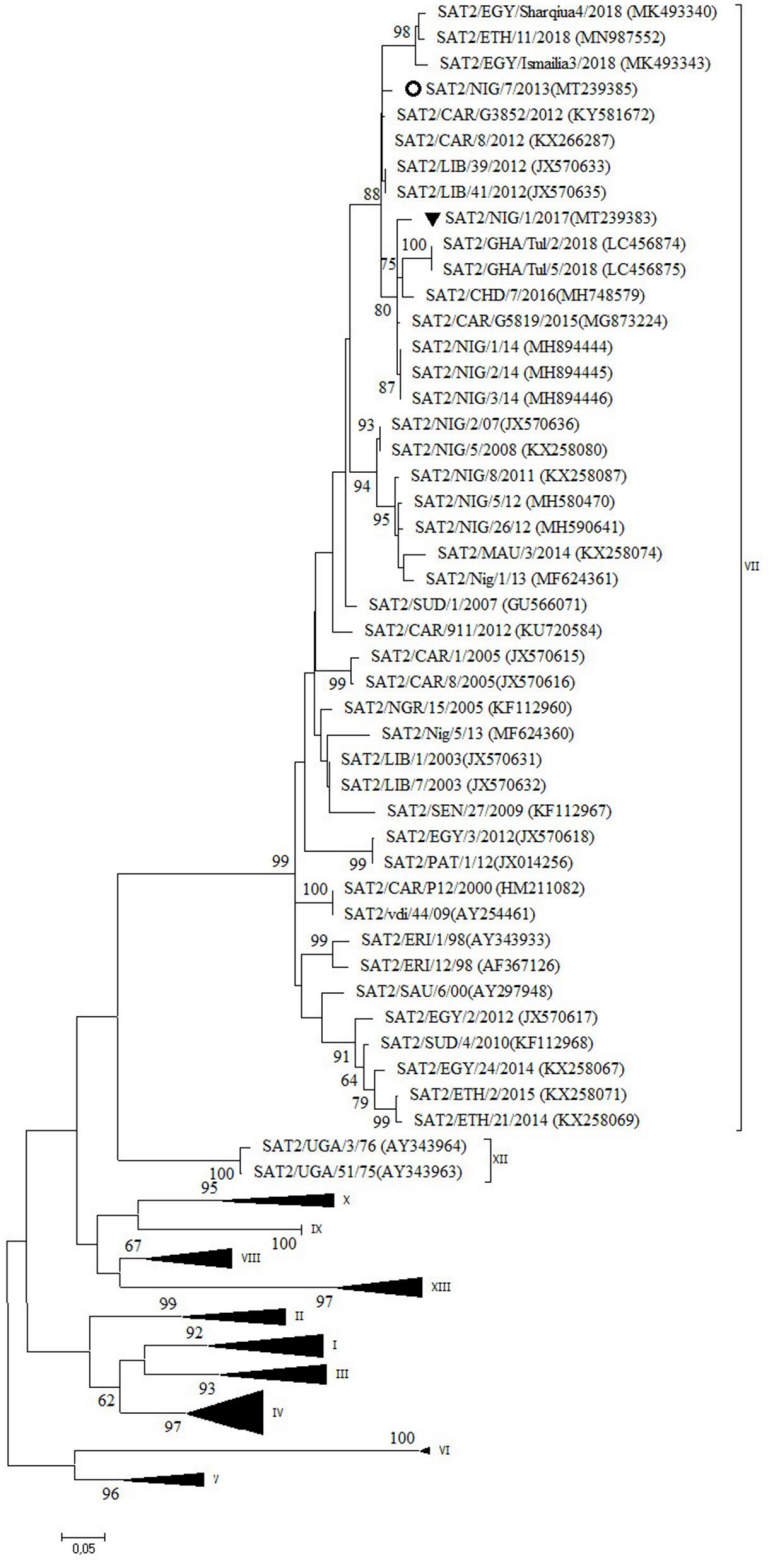
VP1 phylogenetic tree for foot-and-mouth disease virus (FMDV) serotype Southern African Territories (SAT)2 inferred using the maximum likelihood method based on the Tamura-Nei model. Branch lengths indicate the number of substitutions per site. Bootstrap values ≥60% are indicated at the nodes. Novel Nigerian FMDV from this study from Plateau is indicated with ▼ and from Abuja FCT with ⚬.

Two outbreaks, one in Abuja FCT (2013) and one in Plateau State (2017) were serotyped as FMDV SAT2 and further characterized as topotype VII. In the NJ and ML phylogenetic trees (Figure 5), the isolate from 2017 clustered with isolates from 2014 isolated in the neighboring State of Bauchi (14) with 97% VP1 nt identity and with viruses isolated in the neighboring countries CAR and Chad (CHD) in 2015 and 2016, respectively, and in GHA in 2018, with 94–96% VP1 nt identity (28, 31). For the isolate from Abuja FCT (2013), only a partial VP1 sequence was obtained. This isolate showed 95% VP1 nt identity with the isolate from Plateau (2017) and clustered with isolates from neighboring country CAR from 2012 (32, 33) as well as from Libya (LIB) in North Africa from 2012 (34). Both isolates from this study branched separately from a group of Nigerian viruses isolated from 2007 to 2013 and had ≤ 90% VP1 nt identity with this group. Another isolate from Nigeria from 2013, i.e., SAT2/Nig/5/13 (11), clustered with isolates from Libya from 2003 (34).

## Discussion

In the present study, samples were collected from clinical cases of FMD in cattle in Nigeria during a 5-years period. The obtained FMDVs were characterized and compared with previously characterized viruses from Nigeria and neighboring countries. From ~two thirds of the samples, live virus could be isolated on cell cultures, despite the presence of high quantities of FMD viral RNA in most of the other third of the samples. For two outbreaks from 2012, it was not possible to obtain virus or RNA of sufficient quality to allow further characterization. Serotypes O, A, SAT1, and SAT2 were detected by antigen ELISA and later on confirmed by VP1 sequencing. In some cases, a reaction to a second FMDV serotype was observed in the antigen ELISA, particularly to serotype SAT1 in samples positive for serotype A. Although this may suggest a dual infection with two virus strains of different serotypes, we did not identify serotype SAT1 VP1 genomic sequences in samples positive for serotype A.

Two thirds of the investigated outbreaks were in Plateau State (North Central region) where the National Reference Laboratory for FMD is located. And all but two of the outbreaks were in Plateau and surrounding States which are located in the subhumid region of Nigeria and where almost 50% of Nigeria's cattle population can be found (10). Nevertheless, it would be beneficial if more samples could be obtained from regions with lower livestock densities, as well as more samples from small ruminants and wildlife, to get a more complete view on the circulation and distribution of FMDV in Nigeria. This will also be necessary to support a future vaccination-based control plan.

In the present study, three different serotypes of FMDV were observed in 3 years of time in Plateau State: serotype O in 2014, 2015, and 2017, serotype SAT1 in 2015, and serotype SAT2 in 2017. In the same period, serotype A was observed in the neighboring States of Bauchi and Kaduna. Highly remarkable is that in September 2015, in <2 weeks of time, clinical cases of FMD due to serotypes O and SAT1 were observed in Local Government Area (LGA) Jos South in Plateau and due to serotype A in LGA Toro in Bauchi, with both LGAs in close proximity (<100 km). In 2017, serotype A was observed in three remote States [Kaduna (North West), Benue (North Central), and Oyo (South West)] which are separated by several hundreds of kilometers. It has previously been shown that movement of infected livestock is the most important factor in the spread of FMD within endemically infected regions (2, 35, 36). Consequently, the results of the present study suggest that due to the high number of long distance and transboundary cattle movements, FMDV is highly dynamic and widely distributed in Nigeria and illustrate the complex concomitance of FMDV strains in Nigeria.

Half of the investigated outbreaks were FMDV topotype O/EA-3. Based on the phylogenetic trees and nucleotide sequence alignment, the data of the present study suggest the continued circulation in 2017 in Nigeria of the FMDV topotype O/EA-3 virus lineage described by Ehizibolo et al. (11) in 2014 with ~1% VP1 nt change per year. This confirms previous observations from Bertram et al. (32) who suggested a pattern of continuous transmission of FMDV topotype O/EA-3 in the West African region. This % of change in VP1 nucleotide identity is in agreement with previous observations made by Knowles and Samuel (37). The data of the present study also suggest that two different sub-lineages of FMDV topotype O/EA-3 were circulating in Nigeria in 2017, in line with previous observations made by Ehizibolo et al. (14) in 2016. The data of the present study do not allow to conclude whether this is a result of two separate introductions of FMDV topotype O/EA-3 into Nigeria or a result of local virus evolution from a common ancestor. Although in the present study FMDV topotype O/WA was not isolated after 2013, it should be noted that this topotype was isolated in Niger, a country which borders to the north of Nigeria, in 2015 (38) and on a cattle market in Plateau State in 2016 (14). This suggests that the FMDV topotype O/WA continues to circulate in the region despite the abundant presence of clinical outbreaks caused by FMDV topotype O/EA-3.

Two different sub-lineages of FMDV topotype A/Africa lineage G-IV were isolated in Nigeria in 2017, respectively, in Oyo (South West region, bordering Benin) and in Benue and Kaduna (North Central and North West regions, respectively). Based on the available sequence information, the virus isolated in Oyo suggests continued circulation in Nigeria of a virus sub-lineage previously reported in 2009, with 1% change in VP1 nt identity per year, although it seems remarkable that this virus sub-lineage was not detected in Nigeria between 2009 and 2017. It should also be noted that in Benin, a country which borders to the west of Oyo State, the FMD serotype A viruses isolated in 2010 were characterized as topotype A/Africa lineage G-VI while at that time topotype A/Africa lineage G-IV was present in Nigeria (39). The viruses isolated in Benue and Kaduna in the present study may be the result of a new introduction into Nigeria in 2017 as this virus strain did not seem to circulate in Nigeria during the previous years, or at least was not detected.

The present study confirms the occurrence of a newly discovered FMDV topotype SAT1/X (12) in Nigeria in 2015 after an absence of clinical cases of FMD caused by serotype SAT1 for more than 30 years. The latter was further confirmed in the present study by serological testing of 300 samples of sheep and goat and 38 samples from wildlife obtained in the period 2009–2015. None of these 338 serum samples reacted with FMDV serotype SAT1 in the in-house solid-phase competition ELISA (17) performed at Sciensano, whereas antibodies against serotypes A, O, and SAT2 were observed (data not shown). It should however be noted that Dhikusooka et al. (40) could isolate FMDV serotype SAT1 from probang samples from young, healthy, unvaccinated cattle in Uganda. This FMDV strain differed significantly from other SAT1 FMDV strains previously isolated from cattle or buffalo in the same region. This suggests that at least some SAT1 FMDV strains can circulate in cattle herds without giving rise to clinical symptoms. Bastos et al. (41) have described the African buffalo (*Syncerus caffer*) as a reservoir host for the maintenance of FMDV serotype SAT1 and as a source of infection for domestic livestock in Southern Africa. To our interpretation, the role of African buffalo in the etiology of FMD in domestic livestock in West Africa is unclear (42). Two subspecies of the African buffalo, the West African Savannah buffalo (*S. caffer brachyseros*) and the African forest buffalo (*S. caffer nanus*), reside in the respective subhumid and humid border regions between Nigeria and CAR (42). A population of the West African Savannah buffalo is also present in the Yankari National Park in Bauchi (43). This national park is an interface of 2,250 km^2^ between wildlife, domestic animals, and humans and is surrounded by villages populated by farmers and herders. It is located at ~150 km from the first described outbreaks of FMDV topotype SAT1/X in 2015 in LGA Jos South in Plateau, but no studies have ever been conducted to detect FMDV in buffalo or other wildlife species in the Yankari National Park.

The data of the present study suggest the continued circulation in 2017 in Nigeria of the FMD SAT2/VII/Lib-12 virus lineage previously observed in Nigeria in 2014 (14) with ~1% VP1 nt change per year, with a concomitant circulation of this virus lineage in neighboring countries. The data also suggest that three different lineages of FMDV topotype SAT2/VII circulated in Nigeria in 2013. The isolate from Abuja FCT from 2013 seems to be the earliest description of the FMD SAT2/VII/Lib-12 virus lineage in Nigeria, which seems to have become the dominant SAT2/VII virus lineage in Nigeria since then. Similarly, a pattern of repeated introductions of different FMD SAT2/VII virus lineages was observed in neighboring country CAR in the period 2010–2014 (32). The establishment of the FMD SAT2/VII/Lib-12 virus lineage from Libya in Nigeria is another example of the epidemiological link of FMDV that exists between West Africa and North Africa as more recent examples have shown the incursion of FMDV topotypes A/Africa/G-IV and O/EA-3 from West Africa into Algeria in 2017 and 2018, respectively (44, 45).

Taken together, these results indicate that the epidemiology of FMD in Nigeria is dynamic and complex and probably results from a combination of sustained local transmission of present FMDV strains and the incursion of new FMDV strains into Nigeria. This is similar to previous observations made in neighboring country CAR (46). It has been alleged that the incursion of most of these new FMDV strains results from trade of cattle entering Nigeria from neighboring countries (9).

In conclusion, in the present 5-years study conducted in Nigeria in the period 2012–2017, we isolated FMDV of topotypes O/EA-3, O/WA, A/Africa/G-IV, SAT1/X, and SAT2/VII from clinical cases in cattle and compared them with previously obtained FMDVs from Nigeria and neighboring countries. The results of our study suggest that the presence of these FMDVs result from sustained local transmission of FMDV strains present in Nigeria since a number of years ago and from repeated introductions into the country of new FMDV strains with shorter periods of sustained transmission. The epidemiology of FMD in Nigeria is complex, and more studies, including studies in wildlife, are needed to support the implementation of control programs.

## Data Availability Statement

The datasets analyzed during the present study are available from the authors upon reasonable request. The nucleotide sequences obtained in the present study are accessible in GenBank under the numbers indicated in Table 1.

## Ethics Statement

Ethics approval was not required as per institutional guidelines and local legislation as the samples were collected as part of routine investigation of disease outbreaks.

## Author Contributions

HU conceived and designed the study, coordinated laboratory analyses at NVRI, and helped to draft the manuscript. DJL coordinated laboratory analyses at Sciensano, analyzed the data, and drafted the manuscript. AH performed the sequencing studies and the phylogenetic analysis. YW and DDL participated in the design of the study and contributed to sample acquisition. DE helped to coordinate the study and contributed to sample acquisition. AD coordinated safe sample shipment and helped to coordinate the study. KD coordinated and helped to conceive and design the study. All authors contributed to manuscript revision and read and approved the submitted version.

## Conflict of Interest

The authors declare that the research was conducted in the absence of any commercial or financial relationships that could be construed as a potential conflict of interest.

## References

[1] Grubman MJ Baxt B. Foot-and-mouth disease. Clin Microbiol Rev. (2004) 17:465–93. 10.1128/CMR.17.2.465-493.2004 15084510 PMC387408

[2] Di Nardo A Knowles NJ Paton DJ. Combining livestock trade patterns with phylogenetics to help understand the spread of foot and mouth disease in sub-Saharan Africa, the middle east and southeast Asia. Rev Sci Tech. (2011) 30:63–85. 10.20506/rst.30.1.2022 21809754

[3] McElwain TF Thumbi SM. Animal pathogens and their impact on animal health, the economy, food security, food safety and public health. Rev Sci Tech. (2017) 36:423–33. 10.20506/rst.36.2.2663 30152474 PMC6561776

[4] King DP Madi M Mioulet V Wadsworth J Wright CF Valdazo-González B. New technologies to diagnose and monitor infectious diseases of livestock: challenges for sub-Saharan Africa. Onderstepoort J Vet Res. (2012) 79:456. 10.4102/ojvr.v79i2.456 23327376

[5] Freimanis GL Di Nardo A Bankowska K King DJ Wadsworth J Knowles NJ. Genomics and outbreaks: foot and mouth disease. Rev Sci Tech. (2016) 35:175–89. 10.20506/rst.35.1.2426 27217177

[6] Wikipedia Nigeria. Available online at: https://en.wikipedia.org/wiki/Nigeria (accessed April 3, 2020).

[7] Food and Agriculture Organization of the United Nations (FAO) Africa Sustainable Livestock 2050: Transforming livestock sector. Nigeria: What do Long-Term Projections Say? (2019) Available online at: http://www.fao.org/3/CA3374EN/ca3374en.pdf (accessed April 3, 2020).

[8] Alhaji NB Amin J Aliyu MB Mohammad B Babalobi OO Wungak Y. Economic impact assessment of foot-and-mouth disease burden and control in pastoral local dairy cattle production systems in northern Nigeria: a cross-sectional survey. Prev Vet Med. (2020) 177:104974. 10.1016/j.prevetmed.2020.104974 32240887

[9] Fasina FO Connell DR Talabi OA Lazarus DD Adeleke GA Olusanya TP. Foot-and-mouth disease virus strains and examination of exposure factors associated with seropositivity of cattle herds in nigeria during 2007-2009. Prev Vet Med. (2013) 109:334–42. 10.1016/j.prevetmed.2012.10.004 23127691

[10] Lawal-Adebowale OA Livestock production. Chapter 4: dynamics of ruminant livestock management in the context of the nigerian agricultural system. In: Khalid Javed, editor. IntechOpen. (2012) Available online at: https://www.intechopen.com/books/livestock-production/dynamics-of-ruminant-livestock-management-in-the-context-of-the-nigerian-agricultural-system (accessed April 3, 2020). 10.5772/52923

[11] Ehizibolo DO Haegeman A De Vleeschauwer AR Umoh JU Kazeem HM Okolocha EC. Detection and molecular characterization of foot and mouth disease viruses from outbreaks in some states of Northern Nigeria 2013-2015. Transbound Emerg Dis. (2017) 64:1979–90. 10.1111/tbed.12602 28097814

[12] Ehizibolo DO Haegeman A De Vleeschauwer AR Umoh JU Kazeem HM Okolocha EC Foot-and-mouth disease virus serotype SAT1 in cattle, Nigeria. Transbound Emerg Dis. (2017) 64:683–90. 10.1111/tbed.12629 28224715

[13] Ehizibolo DO De Vleeschauwer AR Haegeman A Lefebvre D Nwosuh CI Umoh JU. Serological and molecular epidemiology of foot-and-mouth disease viruses in agro-pastoralist livestock herds in the kachia grazing reserve, Nigeria. Transbound Emerg Dis. (2019) 66:1575–86. 10.1111/tbed.13182 30901506

[14] Ehizibolo DO Fish IH Brito B Bertram MR Ardo A Ularamu HG. Characterization of transboundary foot-and-mouth disease viruses in Nigeria and Cameroon during 2016. Transbound Emerg Dis. (2020) 67:1257–70. 10.1111/tbed.13461 31880066

[15] Ularamu HG Ibu JO Wood BA Abenga JN Lazarus DD Wungak YS. Characterization of foot-and-mouth disease viruses collected in Nigeria between 2007 and 2014: evidence for epidemiological links between West and East Africa. Transbound Emerg Dis. (2017) 64:1867–76. 10.1111/tbed.12584 27718336

[16] Sangare O Bastos AD Venter EH Vosloo W. Retrospective genetic analysis of SAT-1 type foot-and-mouth disease outbreaks in West Africa (1975-1981). Vet Microbiol. (2003) 93:279–89. 10.1016/S0378-1135(02)00439-X 12713891

[17] OIE Foot-and-mouth disease [Chapter 3.1.8]. In: Manual of Diagnostic Tests and Vaccines for Terrestrial Animals 2017. OIE, Paris, France. Available online at: https://www.oie.int/fileadmin/Home/eng/Health_standards/tahm/3.01.08_FMD.pdf

[18] Callahan JD Brown F Osorio FA Sur JH Kramer E Long GW. Use of a portable real-time reverse transcriptase-polymerase chain reaction assay for rapid detection of foot-and-mouth disease virus. J Am Vet Med Assoc. (2002) 220:1636–42. 10.2460/javma.2002.220.1636 12051502

[19] Reid SM Ferris NP Hutchings GH Zhang Z Belsham GJ Alexandersen S. Detection of all seven serotypes of foot-and-mouth disease virus by real-time, fluorogenic reverse transcription polymerase chain reaction assay. J Virol Methods. (2002) 105:67–80. 10.1016/S0166-0934(02)00081-2 12176143

[20] Vandenbussche F Mathijs E Lefebvre D De Clercq K Van Borm S. A tale of tails: dissecting the enhancing effect of tailed primers in real-time PCR. PLoS ONE. (2016) 11:e0164463. 10.1371/journal.pone.0164463 27723800 PMC5056738

[21] Vandenbussche F Lefebvre DJ De Leeuw I Van Borm S De Clercq K. Laboratory validation of two real-time RT-PCR methods with 5'-tailed primers for an enhanced detection of foot-and-mouth disease virus. J Virol Methods. (2017) 246:90–4. 10.1016/j.jviromet.2017.04.014 28457784

[22] Ayelet G Mahapatra M Gelaye E Egziabher BG Rufeal T Sahle M. Genetic characterization of foot-and-mouth disease viruses, ethiopia, 1981-2007. Emerg Infect Dis. (2009) 15:1409–17. 10.3201/eid1509.090091 19788808 PMC2819860

[23] Tamura K Nei M. Estimation of the number of nucleotide substitutions in the control region of mitochondrial DNA in humans and chimpanzees. Mol Biol Evol. (1993) 10:512–26. 10.1093/oxfordjournals.molbev.a040023 8336541

[24] Saitou N Nei M The neighbor-joining method: a new method for reconstructing phylogenetic trees. Mol Biol Evol. (1987) 4:406–25. 10.1093/oxfordjournals.molbev.a040454 3447015

[25] Felsenstein J. Confidence limits on phylogenies: an approach using the bootstrap. Evolution. (1985) 39:783–91. 10.1111/j.1558-5646.1985.tb00420.x 28561359

[26] Tamura K Stecher G Peterson D Filipski A Kumar S. MEGA6: Molecular evolutionary genetics analysis version 6.0. Mol Biol Evol. (2013) 30:2725–9. 10.1093/molbev/mst197 24132122 PMC3840312

[27] Ludi A Ahmed Z Pomeroy LW Pauszek SJ Smoliga GR Moritz M. Serotype diversity of foot-and-mouth-disease virus in livestock without history of vaccination in the Far North region of Cameroon. Transbound Emerg Dis. (2016) 63:e27–38. 10.1111/tbed.12227 24735162 PMC4499489

[28] Bertram MR Delgado A Pauszek SJ Smoliga GR Brito B Stenfeldt C. Effect of vaccination on cattle subclinically infected with foot-and-mouth disease virus in cameroon. Prev Vet Med. (2018) 155:1–10. 10.1016/j.prevetmed.2018.04.003 29786519

[29] Teye MV Sebunya TK Fana EM King DP Seoke L Knowles NJ. Foot-and-mouth disease in southern Ghana: occurrence and molecular characterization of circulating viruses. Trop Anim Health Prod. (2019) 51:1667–77. 10.1007/s11250-019-01864-8 30879248

[30] Ehizibolo DO Perez AM Carrillo C Pauszek S AlKhamis M Ajogi I. Epidemiological analysis, serological prevalence and genotypic analysis of foot-and-mouth disease in Nigeria 2008-2009. Transbound Emerg Dis. (2014) 61:500–10. 10.1111/tbed.12054 23347819

[31] Abdel-Aziz AI Romey A Relmy A Gorna K Laloy E Métras R. Seroprevalence and molecular characterization of foot-and-mouth disease virus in Chad. Vet Med Sci. (2020) 6:114–21. 10.1002/vms3.206 31845545 PMC7036305

[32] Bertram MR Bravo de Rueda C Garabed R Dickmu Jumbo S Moritz M Pauszek S. Molecular epidemiology of foot-and-mouth disease virus in the context of transboundary animal movement in the Far North region of cameroon. Front Vet Sci. (2018) 5:320. 10.3389/fvets.2018.00320 30619901 PMC6301994

[33] Lycett S Tanya VN Hall M King DP Mazeri S Mioulet V. The evolution and phylodynamics of serotype A and SAT2 foot-and-mouth disease viruses in endemic regions of Africa. Sci Rep. (2019) 9:5614. 10.1038/s41598-019-41995-4 30948742 PMC6449503

[34] Ahmed HA Salem SA Habashi AR Arafa AA Aggour MG Salem GH. Emergence of foot-and-mouth disease virus SAT 2 in Egypt during 2012. Transbound Emerg Dis. (2012) 59:476–81. 10.1111/tbed.12015 23025522

[35] Rweyemamu M Roeder P Mackay D Sumption K Brownlie J Leforban Y. Epidemiological patterns of foot-and-mouth disease worldwide. Transbound Emerg Dis. (2008) 55:57–72. 10.1111/j.1865-1682.2007.01013.x 18397509

[36] Knight-Jones TJD McLaws M Rushton J. Foot-and-mouth disease impact on smallholders – what do we know, what don't we know and how can we find out more? Transbound Emerg Dis. (2017) 64:1079–94. 10.1111/tbed.12507 27167976 PMC5516236

[37] Knowles NJ Samuel AR. Molecular epidemiology of foot-and-mouth disease virus. Virus Res. (2003) 91:65–80. 10.1016/S0168-1702(02)00260-5 12527438

[38] Souley Kouato B Elliot FM King DP Hyera J Knowles NJ Ludi AB. Outbreak investigations and molecular characterization of foot-and-mouth disease viruses circulating in south-west Niger. Transbound Emerg Dis. (2018) 65:146–57. 10.1111/tbed.12642 28345819

[39] Gorna K Houndjè E Romey A Relmy A Blaise-Boisseau S Kpodékon M. First isolation and molecular characterization of foot-and-mouth disease virus in Benin. Vet Microbiol. (2014) 171:175–81. 10.1016/j.vetmic.2014.03.003 24720890

[40] Dhikusooka MT Ayebazibwe C Namatovu A Belsham GJ Siegismund HR Wekesa SN. Unrecognized circulation of SAT 1 foot-and-mouth disease virus in cattle herds around queen elizabeth national park in Uganda. BMC Vet Res. (2016) 12:5. 10.1186/s12917-015-0616-1 26739166 PMC4704403

[41] Bastos AD Haydon DT Forsberg R Knowles NJ Anderson EC Bengis RG. Genetic heterogeneity of SAT-1 type foot-and-mouth disease viruses in southern Africa. Arch Virol. (2001) 146:1537–51. 10.1007/s007050170077 11676416

[42] Di Nardo A Libeau G Chardonnet B Chardonnet P Kock RA Parekh K. Serological profile of foot-and-mouth disease in wildlife populations of west and central Africa with special reference to Syncerus caffer subspecies. Vet Res. (2015) 46:77. 10.1186/s13567-015-0213-0 26156024 PMC4495843

[43] Bergl RA Dunn A Haruna S Mshelbwala J Nyanganji G Aerial survey of elephants and other large mammals at Yankari Game Reserve, Bauchi State, Nigeria. (2011) Available online at: http://africanelephantdatabase.org/system/population_submission_attachments/files/000/000/118/original/svyFWNGYKR2011AT-1.pdf (accessed April 7, 2020).

[44] WRLFMD FAO World Reference Laboratory for Foot-and-Mouth Disease. Genotyping Report, 10th July 2018. BATCH: WRLFMD/2018/00019 Available online at: https://www.wrlfmd.org/sites/world/files/WRLFMD-2018-00019-ALG-GTR-O-O_001.pdf (accessed April 15, 2020).

[45] Pezzoni G Bregoli A Grazioli S Barbieri I Madani H Omani A. Foot-and-mouth disease outbreaks due to an exotic virus serotype A lineage (A/AFRICA/G-IV) in Algeria in 2017. Transbound Emerg Dis. (2019) 66:7–13. 10.1111/tbed.13017 30222914

[46] Pomeroy LW Bjørnstad ON Kim H Jumbo SD Abdoulkadiri S Garabed R. Serotype-specific transmission and waning immunity of endemic foot-and-mouth disease virus in cameroon. PLoS ONE. (2015) 10:e0136642. 10.1371/journal.pone.0136642 26327324 PMC4556668

